# Supplementary material for: Are carbon emissions trading and green financial instruments synergistic? -Comprehensive quantitative research based on content analysis
Source: PLoS One. 2024 Mar 7;19(3):e0298601. doi: 10.1371/journal.pone.0298601 (PMC10919601; doi:10.1371/journal.pone.0298601)
Supplement: S1 Data — (ZIP) [file pone.0298601.s001.zip › Data/地级市Z_F工作报告.zip!/┴╔─■/╕╖╨┬╩╨/└┤╫╘╣1⁄2╓┌║┼í╛┬φ┐╦╩2╛▌═°í┐_2006.docx]

各位代表：

　　现在，我代表市人民政府向大会报告工作，请予审议，并请各位政协委员提出意见。

　　一、２００６年工作回顾

　　过去的一年，在市委的正确领导下，全市上下紧紧围绕推进转型振兴和构建和谐阜新两大主题，开拓进取，扎实工作，克服了特大旱灾等不利因素的影响，基本完成了市十三届人大四次会议确定的目标和任务，经济运行质量和效益明显提高，各项社会事业有新的发展。

　　预计全市生产总值完成１５８．４亿元，增长１０．９％，其中，第一产业下降５．１％，第二产业增长１４．７％，第三产业增长１７．４％。全社会固定资产投资９５亿元，增长３０％。地方财政一般预算收入８．９６亿元，增长２０．６％。社会消费品零售总额７５亿元，增长１４．３％。实际直接利用外资１９３２万美元，增长４５．５％。城镇居民人均可支配收入７６００元，增长１４．２％；农民人均纯收入２８５０元，下降７．８％。市区街巷路和城市道路改造工程、农村义务教育扶困助学工程等为全市人民办的１４件实事全部完成。

　　（一）积极抢抓新的发展机遇，经济转型迈出重大步伐。经过积极争取，国家和省对阜新经济转型试点工作给予了特殊政策支持，赢得了转型试点以来的第二次重大发展机遇。国务院办公厅以办９９６号件形式对阜新经济转型从产业政策和重大项目建设等８个方面提出了明确支持意见；省委、省政府下发了《关于进一步做好阜新市经济转型试点工作的意见》，设立了阜新经济转型专项资金，确定了土地、开放、产业发展等５个方面的支持政策。全市上下积极抢抓机遇，加大政策跟踪落实力度，为转型振兴创造了良好条件。全市产业结构进一步调整优化，“三大产业基地”建设迈出新步伐。农产品及食品加工业占全市规模以上工业比重上升，新型能源基地建设稳步推进，煤化工产业基地建设开始起步；装备制造、新型建材、精细化工、新型电子、玛瑙加工、北派服饰等“六大优势特色产业”不断发展壮大。城乡基础设施建设进一步加强，生态环境明显改观。就业再就业、棚户区改造和采煤沉陷区治理等民生工程取得显著成效，转型给人民群众带来了实实在在的利益。全市上下精神振奋，更加充满信心地投身于转型振兴的宏伟大业。

　　（二）深入开展“工业年”活动，经济运行质量明显提高。规模以上工业实现总产值１５１亿元，增长３０％，利润２．３亿元，同比减亏增盈２．６亿元。实施技改项目１１０项，争取省技改贴息资金８０５０万元；引进建设销售收入亿元以上项目２０个。新增销售收入超亿元企业１０户，达到３０户。阜新双汇、伊利等一批农产品加工企业进一步发展壮大。阜新发电公司三期１台３５万千瓦机组、金山热电２台１５万千瓦机组试运行，华能风力发电一期工程、彰武火电项目达到核准条件。阜矿集团内蒙古白音华露天煤矿开工。总投资４００亿元、建设规模５２０万吨甲醇及深加工产品的煤化工项目被列入辽宁省产业基地和全国煤化工发展规划，前期工作进展顺利。横跨两省区、９个旗县，总投资５４．５亿元的巴新铁路项目达到国家核准条件，投资协议正式签订。阜矿集团总收入突破３５亿元，对全市经济发展作出贡献。阜新发电公司发电近４０亿度，实现较大幅度增长。阜新供电公司售电量首次超过２０亿度。全市新增规模以上工业企业３７户，达到２６０户。民营经济占全市经济总量比重进一步提高，实缴税金５．７亿元，增长２６．１％。

　　园区建设步伐加快，阜新经济开发区基础设施建设进一步完善，国家农业科技园区被省政府批准为省级高新技术产业园区，平西、城南、阜蒙县等５个工业园区２４０万平方米土地达到企业入驻条件。实施了矿井水、粉煤灰综合利用等循环经济项目，田园实业等４户企业和清河门工业园区被确定为省第一批循环经济试点企业和工业园区。万元地区生产总值能耗下降４．７％，完成省考核指标。[Page]

　　（三）加快推进农业结构调整，社会主义新农村建设实现良好开局。今年我市遭受了百年不遇的特大旱灾。面对旱情的严峻考验，全市上下团结一心，奋起抗灾自救。各级干部靠前指挥，因地制宜地采取了浇水抗旱、发展养殖业、建设蔬菜保护地和劳务输出等措施，使灾害损失降到了最低程度。全市畜牧业发展取得新突破，猪、牛、羊、禽饲养量分别增长１１．３％、１０．８％、１４．９％和１３．４％，肉羊、白鹅人均饲养量居全省首位，生猪产业成为全省最完整的产业链条，奶牛饲养量、牛奶产量居全省前列。推进“百万农民增收致富工程”，新增项目户５．５万户。输出农村劳动力２３．８万人。新建日光温室和冷棚１．５万栋，增长３．７倍，实现历史性突破；完成青黄贮５．３万窖，居全省首位。加强生态环境建设，植树造林和封山育林７４万亩。东北黑土区水土流失综合治理试点工程通过国家验收。机械化保护性耕作、节水灌溉和人工增雨在抗旱中发挥了积极作用。农村综合改革不断深化，税费改革成果进一步巩固，集体林产权制度改革全面推进。

　　以县乡工业化、生态与基础设施建设等“八大工程”为载体，全面启动社会主义新农村建设，在２个乡镇３０个村开展试点并取得初步成果。县域经济发展加快，阜蒙县规模以上工业总产值增长８０％以上，彰武县地方财政一般预算收入增长３０％以上。整合财力，投入专项资金和抗旱救助资金２．２亿元；落实惠农政策，发放粮食直补、良种补贴、农机具补贴１．１亿元。

　　（四）加大招商引资工作力度，对外开放再创新水平。把招商引资作为推动地区经济发展的战略性举措，进一步完善了招商引资各项政策措施。参加省政府组织的香港经贸洽谈会、沈阳韩国周等８次境内外招商活动，对接洽谈了一批合资合作项目。组织１５０多个招商小分队、５００余人次赴南京、温州、香港、日本等境内外重点地区招商，取得明显成效。成功举办首届“中国·阜新玛瑙节”经贸洽谈活动和“全国民营企业资源型城市转型阜新行”项目推介活动，增强了阜新对外吸引力和影响力。杰生铜业、中亿实业等５个利用外资项目资金到位额均超过１００万美元。总投资２亿元的山东泰和集团纸面石膏板等一批内资项目开工建设。全市招商引资实际到位４６．３亿元，增长２２％。全年出口创汇４０００万美元，增长１４．５％。

　　（五）不断深化各项改革，国企改革迈出新步伐。把改革作为促进经济发展的根本动力，完成各类国有企业转制１０８户。国有工业企业改制任务全面完成，改制后企业活力明显增强。公用事业改革取得突破，香港百江燃气公司对市煤气公司进行了战略重组。商贸流通企业和粮食企业改制取得进展，大商千盛百货租赁百货大楼运营良好。采取兼并、破产等形式处理“壳企业”３３户。组建了国有资产监督管理委员会，加强对国有资产的有效监管。贯彻《公务员法》，公务员工资制度改革和事业单位收入分配制度改革全面启动。

　　（六）突出发展现代服务业，第三产业发展步伐加快。国美电器落户阜新，蔬菜批发市场开工建设，容大时代广场主体工程竣工。推进了“万村千乡”市场工程建设，新增各类连锁店２００余家。启动“中国优秀旅游城市”创建工作，完成了《阜新旅游发展总体规划》编制工作。海棠山旅游基础设施国债项目竣工，瑞应寺恢复性建设步伐加快。海州矿环境综合治理一期工程开工，国家矿山公园建设示范工程全面启动。全市旅游业总收入６．８亿元，增长３１．５％。房地产业稳步发展，全年开发住宅面积２５６万平方米。以便民服务为核心的社区服务业发展步伐加快。中介服务、邮政、通讯等行业取得了新进展。烟草、石油等中省直单位为地方经济发展作出了重要贡献。

　　（七）积极做好财税金融工作，为经济社会发展提供了资金保障。财政等部门加大资金争取力度，审计部门充分发挥审计监督职能作用，加强资金调度和管理，确保了重点支出。全市国税收入突破１０亿元，增长２２．６％；地税收入超过８亿元，增长２３．３％。全市金融机构各项贷款余额１７７．７亿元，比年初增加１８．１亿元，增长１１．２％。市农发行、工行、中行、建行等金融机构为经济社会发展作出了积极贡献。农村信用社投放支农贷款１６．９亿元，有力地支持了新农村建设和抗旱救灾工作。市商业银行对地方工业企业和重点项目投放贷款余额比年初净增７．１亿元，增长４４．５％。[Page]

　　（八）大力加强基础设施建设，城乡面貌发生显著变化。沈阳至彰武、铁岭至阜新、阜新至朝阳三条高速公路开工建设，彰武至通辽高速公路完成征地动迁核量。阜新至北京直通旅客列车开通。阿金公铁立交桥竣工通车。完成创业路、海新路等１６条街巷路和城市道路维修改造。细河５号坝竣工，细河小兰桥、太平区红玛瑙广场投入使用。我市被国家建设部评为“水环境治理优秀城市”，细河治理工程被国家授予“中国人居环境范例奖”。实施地下管网改扩建工程，新增供热面积２４１万平方米、煤气用户１．２万户。投入１．６亿元建设的城市污水处理厂试运行，中水回用工程开工建设。引白水源工程实现投资１．５亿元，完成３８公里管线铺设任务。城市绿化覆盖率达到３９．６％，人均占有公共绿地面积达到７．３平方米，我市被评为“辽宁省园林城市”。开展“城市管理提高年”活动，加大了城市环境治理和保护工作力度。城市大气环境进一步改善，全年２／３以上天数空气质量达到国家二级标准。完成通村油路３６５．８公里，彰武县城出口路、东环路改造工程完工。阜蒙县十家子镇等重点乡镇规划进一步完善。

　　动员全社会力量，克服各种困难，提前一年基本完成全省规模最大、任务最重的棚户区改造工程。两年共投资３０亿元，拆除房屋２２１万平方米，新建住宅２７２万平方米，７０７栋新楼拔地而起，７６６９６户２０余万棚户区居民喜迁新居，困扰城市南部地区居民多年的吃水难、行路难、入厕难等诸多问题得到有效解决。棚户区改造工程在创造“阜新棚改速度”的同时，也培育了以高度负责、知难而进、团结协作、扎实苦干为核心内容的“阜新棚改精神”。采煤沉陷区治理一期工程基本完工，１８３３３户沉陷区居民得到安置。

　　（九）认真解决关系人民群众切身利益的现实问题，和谐阜新建设扎实推进。坚持以人为本，高度关注民生。以促进零就业家庭、沉陷区和棚户区居民等困难群体再就业为重点，建立就业援助长效机制，加大就业帮扶力度，全市实现就业和再就业７．６万人，实现零就业家庭动态为零。工、青、妇等群团组织在安置就业中发挥了积极作用。社保体系进一步完善，基本养老保险实现了市级统筹，各类社会保险覆盖面进一步扩大，启动了未参保国有困难企业退休人员医疗保险。提高了８．３万名企业离退休人员待遇、１７．７万名城市低保及临时救助标准和６９７９名优抚对象抚恤标准，实施了城市低保人员大病医疗救助制度。

　　社区卫生服务体系进一步完善，卫生服务覆盖率达到９８．１％。实施“蓓蕾生命救助行动”，对７３名低保家庭患儿进行了医疗救助；开展慈善义工活动，大力营造团结友爱、互助文明的社会风气。新型农村合作医疗全面启动，农业人口参合率达到８０％。农村中小学办学条件进一步改善，完成农村危险校舍改造２．９万平方米，建设九年寄宿制学校４所。全市农村义务教育阶段学生全部免交杂费，对１４．６万名城乡困难家庭学生进行了资助。开展扶贫帮困，全力做好受灾农民救助工作。以产业扶贫为重点，对６８个重点贫困村实施整村扶贫开发。全市农村实现户户通电。

　　（十）大力发展科技教育，各项社会事业全面进步。加强与高等院校、科研院所合作，研发中心建设、高新技术产品开发、科技成果转化取得较大进展。全市实现高新技术产业产值１８亿元，增长２０％，有１５户企业通过省级高新技术企业认证。引进推广农业新品种１１０个、新技术２５项。辽工大积极为阜新经济转型提供科技支持和人才支撑。推进素质教育，新一轮“普九”工作取得新进展。示范性普通高中公费生招生比例大幅度提高，促进了教育均衡发展。中等职业教育改革取得积极成果，市技工学校通过“国家高级技校”评估验收。全年职业教育招生９２７５人，就业率达９５％，创历史最好水平。“一村一名大学生”工程累计培养８６４人。引进国外智力工作取得明显成效，聘请国外专家２６人。人力资源大厦交付使用，在促进人才开发和劳动力就业中发挥了重要作用。市中医院通过国家中医药“十五”重点专科项目建设评审验收。４个省级乡镇文化中心、市篮球广场等一批公益设施和阜蒙县３１家乡镇卫生院改扩建工程投入使用。“阜新玛瑙雕”被列入国家首批非物质文化遗产保护名录。城乡群众文化活动、全民健身活动蓬勃开展。农村有线电视村村通工程基本完成，市区和阜蒙县、新邱区有线电视实现光缆连接。农村部分计划生育家庭奖励扶助试点工作受到国家表彰。我市顺利通过省级卫生城复检。住房公积金贷款在解决城市中低收入家庭住房方面发挥了重要作用。关爱老龄事业，重视残疾人工作，加强了红十字会组织建设。[Page]

　　（十一）加强社会主义精神文明和民主法制建设，政府自身建设取得新成效。以“知荣辱、树新风、促转型”为主题的群众性道德实践活动广泛开展，和谐村屯、和谐社区等精神文明创建活动取得新成效。自觉接受人大依法监督和政协民主监督，及时办理人大代表议案、建议和政协提案，办复率１００％，满意率９７％。社会治安防控体系建设不断完善，打黑除恶、打击传销等专项行动取得明显成效，人民群众安全感进一步增强。“五五”普法启动实施。行政执法监督工作得到加强。

　　进一步提高办事效率，共有３０６项行政许可及非行政许可审批项目进入行政服务大厅。全市受理市民投诉３．２万件，办结率８７．５％。通过市长公开电话妥善处理市民反映问题３３６件。认真做好信访工作，实行市长接待上访制度，及时化解了各类不稳定因素。加强廉政建设和行政效能建设，查处商业贿赂案件２６件，对棚户区改造等１１项重大建设项目开展行政效能监察，加大了行政责任追究。通过《行风热线》为群众和企业解决难题７５５件。全面落实安全生产责任制，强化专项整治和基础管理，安全生产形势总体保持平稳。

　　认真落实党的民族政策，重视和支持民族地区经济社会发展，民族地区资源开发、特色产业发展步伐加快。阜蒙县胡尔沁说书被列入国家非物质文化遗产保护名录。依法加强宗教事务管理，对海州庙周边环境进行了整治。

　　工商、质量技术监督、出入境检验检疫局等省直部门在支持地方经济发展中发挥了重要作用。统计、物价、消防、档案、地方志、地震监测、外事侨务、对台事务、国家安全、国防教育、民防、民兵预备役等工作进一步加强。

　　各位代表，在“十一五”开局之年，我市经济社会发展取得新的成绩，阜新转型振兴进入一个新的发展阶段。这是在市委的领导下，在市人大、市政协的监督支持下，全市各族人民团结奋斗、开拓进取、顽强拼搏的结果。在此，我代表市人民政府向为阜新转型振兴和构建和谐阜新付出辛勤劳动的全市各族人民，向给予政府工作大力支持的人大代表、政协委员和各民主党派、工商联、无党派人士、人民团体，向为阜新经济建设作出积极贡献的中省直单位、驻阜部队、武警官兵、公安干警，向所有关心、支持阜新发展的各界人士、海内外朋友表示衷心的感谢并致以崇高的敬意！

　　在取得成绩的同时，我们也清醒地认识到，阜新在经济社会发展中还面临着一些亟待解决的矛盾和问题。主要表现在：经济总量小，结构性矛盾突出，财政仍然十分困难；农业基础设施薄弱，抗灾能力不强；经济增长方式粗放，资源利用率低，煤矿安全生产形势严峻；下岗职工多，就业再就业稳定性较差，部分群众生活还很困难；在政府机关中，还不同程度地存在工作责任感不强，服务意识不够，执行力不高等问题，经济发展软环境尚需进一步优化。这些矛盾和问题，我们要在今后工作中认真加以解决。

　　二、２００７年工作的总体要求和主要任务

　　今年是加快推进阜新转型振兴的关键一年，也是为完成“十一五”规划奠定基础的关键一年，做好今年的各项工作，具有特殊重要的意义。我们要立足关键之年，突出项目意识，坚持科学发展，进一步增强工作的责任感、紧迫感和危机感，动员全社会力量，凝聚各方面智慧，解放思想，更新观念，抓住机遇，开拓进取，努力实现各项工作的突破性进展，全面加快转型振兴和构建和谐阜新进程。这是我们神圣的历史使命，也是全市人民的殷切希望。

　　２００７年政府工作的总体要求是：高举邓小平理论和“三个代表”重要思想伟大旗帜，坚持以科学发展观统揽全局，围绕推进转型振兴和构建和谐阜新两大主题，突出建设“三大产业基地”和培育“六大优势特色产业”，着力抓好“工业年”活动、社会主义新农村建设和“友爱阜新”、“平安阜新”建设，大力推进改革开放和科技创新，加快经济结构调整，转变经济增长方式，努力解决民生问题，实现国民经济和社会事业又好又快发展。[Page]

　　今年经济和社会发展主要目标是：全市生产总值实现１９０亿元，增长１８％，力争突破２００亿元，增长２０％以上；全社会固定资产投资突破１００亿元，增长１５％以上；地方财政一般预算收入突破１０亿元，增长１６％以上；社会消费品零售总额８６亿元，增长１４．７％；直接利用外资２４５０万美元，增长２６．８％；出口创汇４８００万美元，增长２０％；城镇居民人均可支配收入８５００元，增长１１．８％；农民人均纯收入３５００元，增长２２．８％；城镇登记失业率控制在５．５％以内；万元地区生产总值能耗降低５％。

　　（一）加快推进转型振兴，确保“工业年”活动取得更大成果

　　实施工业强市战略，坚持走新型工业化道路，转变增长方式，提高发展质量，实现地区产业结构优化升级。

　　推进“三大产业基地”建设，加快经济结构调整。实施项目攻坚战，新开工建设投资千万元以上项目１０５个，其中销售收入亿元以上项目２０个。加快煤化工项目前期工作，尽快实现项目核准，一期工程尽早开工。支持阜矿集团、阜新发电公司、春成集团等骨干企业做大做强，阜新发电公司三期、金山热电项目达产达效，阜矿集团内蒙古白音华煤矿正式投产，华能风力发电一期工程竣工，巴新铁路项目上半年开工建设，力争彰武火电项目开工。加大煤层气、地热、风能、太阳能、生物质能等新型能源的开发力度。支持阜新双汇、伊利等重点企业增资扩产，推进一批农产品及食品工业重大项目建设。围绕壮大“六大优势特色产业”，加大对现有核心企业支持力度，引导企业实施一批资源消耗低、环境污染少、人力资源得到充分发挥的新型产业项目。全年新增销售收入超亿元企业１０户，达到４０户；规模以上工业企业新增４０户，达到３００户。

　　大力发展高新技术产业，促进产业发展模式由传统产业向高新技术产业转变、资源利用方式由初加工向深加工转变。重点支持拥有自主知识产权和自有品牌、有较好市场前景的企业，加快用先进适用技术推动产品和产业结构优化升级，延长优势产业链条，培育产业集群。全年实施技改项目１３０个，完成技改投入２８亿元。认真做好循环经济试点工作，推进循环型加工企业和工业园区建设。要正确处理好项目建设与环境保护的关系，加强企业资源消耗定额管理和对污染物排放限制，全面推行清洁生产。搞好粉煤灰、煤矸石、矿井水等工业废弃物综合利用和医疗垃圾无害化处理，实施生活垃圾资源化利用。实现经济社会健康协调可持续发展。

　　加快各类园区建设，推进生产要素集聚。科学布局产业，突出特色优势，明确园区定位，高起点规划园区。多方筹措资金，加大对各类园区基础设施投入力度，加快完善园区功能，使各类园区尽快达到企业入驻条件。经济开发区和高新技术产业园区要充分发挥对外开放先导区和示范区作用，提高入区项目质量，提升单位面积投资密度和产出效益，在园区发展和项目建设上实现新的突破。

　　（二）坚持以发展现代农业为着力点，扎实推进社会主义新农村建设

　　以社会主义新农村建设统领“三农”工作全局，发挥比较优势，壮大县域经济，在农村经济增长、社会进步、环境改善等方面取得新的进展。

　　大力发展现代农业。以工业化理念谋划农业，调整优化农村经济结构，推动农业产业化经营。做大做强畜牧业，培育发展龙头企业，抓好生猪、奶牛、白鹅、肉羊、肉鸡等配套基地建设。重点发展一批畜牧业饲养小区和专业大户，促进畜牧业饲养方式的转变。进一步加强重大动物疫病防控体系和良种繁育体系建设。加大种植业内部结构调整力度，提高农业综合生产能力。以“一村一品”为切入点，优化农产品布局，培育优势特色产业。抓好“四位一体”生态户和蔬菜大棚等设施农业建设，加快发展节水农业，继续扩大小杂粮、花生、食用菌、“三杏”等特色种植业和果业生产。启动实施“生态家园富民行动”，发展农业循环经济。[Page]

　　千方百计增加农民收入。积极扶持龙头企业和配套基地建设，发挥农产品基地在农民增收中的重要作用。大力发展农民专业合作组织，增强农村集体经济组织服务功能。引导和扶持一批种养殖能手、科技示范户、生产经营大户，带动农民增收。加强农民职业技能培训，加大劳务输出力度，全年输出农村劳动力１９万人。深入推进“百万农民增收致富工程”，全年新增项目户５万户。

　　加强农业基础设施和生态环境建设。大力推进节水灌溉、蓄水集水等工程建设，提高农业抗灾能力。实施东北黑土区综合防治一期工程建设，搞好水土流失综合治理。巩固发展退耕还林成果，推进防沙治沙、农田林网、村屯绿化等重点工程建设，完成植树造林和封山育林７０万亩。提高农业机械化水平，扩大机械化保护性耕作面积。扩建人工增雨基地，提高增雨蓄水能力。统筹规划，突出重点，着力推进一批中心村镇建设。加强农村环境建设，搞好村容村貌治理。

　　推进农村综合改革。搞好土地延包确权，依法推进多种形式的土地使用权流转和适度规模经营。做好小型水利产权制度和集体林产权制度改革工作。进一步改善金融服务环境，开展农业保险试点工作。落实支农惠农政策，整合财力资源，加大对“三农”的投入力度。抓好试点，扎实推进社会主义新农村建设。

　　（三）大力发展现代服务业，培育经济转型新的增长点

　　以扩大规模、调整结构、提高水平、形成网络为目标，推动服务业发展。做好东市场等商业区改造规划，集聚建材、家具市场，改扩建果品市场、旧物市场，开工建设粮食市场，完成蔬菜批发市场二期工程建设。容大时代广场投入运营。推进“万村千乡”市场建设工程，形成覆盖农村的市场服务网络。大力发展现代物流业，规划建设综合性、专业性、区域性物流中心。加快发展房地产业，规范发展信息、咨询、评估等各类中介组织。坚持统筹规划、分步实施，搞好玛瑙城建设。充分发挥藏传佛教文化等特色资源优势，包装和实施一批文化旅游产业项目。要积极引导扶持乡村旅游业的发展。海棠山景区要进入国家４Ａ级景区行列。搞好海州矿国家矿山公园建设，打造世界级工业遗产旅游项目。力争实现“中国优秀旅游城市”创建目标。

　　积极改善金融环境，搭建银企对接平台。推进诚信体系建设，做好金融机构业绩评价工作。加强与省开发行、农发行等政策性银行及其它股份制银行的合作。加快培育资本市场，争取企业上市工作实现突破。组建中小企业担保中心，解决民营企业贷款难问题。推进市商业银行增资扩股，加快城市信用社分类处置改革。

　　千方百计增加财政收入。整顿税收秩序，加强税收级次管理和对重点税源的监控。要依法加强税收和非税收入征管，打击偷、逃税等违法行为，确保税收及时足额均衡入库。继续完善预算制度，整合财力资源，推进公共财政体系建设，提高财政资金使用的规范性、安全性、有效性。优化配置财力资源，将财政资金集中用于保工资、保运转、保稳定和公共项目投入。加强国库集中支付和政府集中采购工作，启动县级国库集中支付改革试点。要厉行增收节支、开源节流，强化财政管理，最大限度地减少跑冒滴漏，实现财政资金效益最大化。加强对重点支出的审计监督和财政监督。

　　（四）深化招商引资工作，全面提高对外开放水平

　　牢固树立大开放理念，充分发挥我市的政策优势和资源优势，坚持以项目为核心，抓住国际产业转移和国内南资北移机遇，积极扩大招商引资，在更广阔领域、更大范围配置资源，实现我市对外开放工作的新突破。

　　围绕“三大产业基地”和“六大优势特色产业”，推进重点企业、城市基础设施以及商贸、文化、公用事业等领域的招商引资。立足我市实际，加强项目开发，包装一批高质量的项目，增强对投资者的吸引力。突出江苏、浙江、香港、韩国、新加坡等境内外重点地区，创新招商方式，坚持专业小分队招商和高层推动招商相结合，采取亲情招商和网上招商等多种形式，引进一批知名企业落户阜新，同时引进技术、人才和先进理念。充分发挥阜矿集团等重大骨干企业的规模优势，整合资源，扩大产业招商，实行强强联合。要提高招商效率和质量，明确责任主体，加强督查考核，完善政策措施。要抓好重大项目的跟踪落实，推进项目签约落地。强化招商项目全程服务，积极营造“亲商、安商、富商”的商务环境，提高聚集生产要素的能力。[Page]

　　加强与沈阳的经济合作，融入沈阳经济圈；利用辽西沿海经济区的优势，抢抓辽宁“五点一线”开发的机遇，在区域经济格局中谋求合作共赢，实现借力发展。

　　积极发展对外贸易，进一步拓宽国外市场。把握国际市场需求，调整出口产品结构，大力支持重点出口企业扩大规模。以争取国外无偿援助项目和劳务输出为重点，进一步开展对外经济技术合作。

　　（五）加快体制机制创新，不断增强经济发展活力

　　巩固国有企业改革成果，解决好工业企业改制遗留问题，健全法人治理结构。抓住阜矿集团对阜橡公司战略重组机遇，积极探索国企改革新路。加快推进商贸流通、建筑、交通运输、公益性和特殊行业及粮食企业等各类国有企业改革，全年完成２０户。认真做好各项准备，推进厂办大集体改革进程。做好氟化学总厂等５户企业政策性破产和“壳企业”依法破产实施工作。坚持职代会制度，切实维护转制企业职工的合法权益。用市场机制整合城市各类资源，加大经营城市力度，最大限度地盘活城市存量资产。整合经营性、非经营性和资源性国有资产，建立健全国有资产监督管理办法，形成有效的监管体系，增加国有资产收益，避免国有资产流失。

　　加大对民营经济的支持力度。落实各项政策措施，进一步优化民营经济发展的市场、政策、法治、服务环境。集中力量支持一批现有民营骨干企业发展壮大，形成规模效应和竞争优势。制定鼓励政策，启动和激活民间资本，激励全民自主创业，增强社会创造力。引导民营企业加强制度创新、管理创新和科技创新，提高企业综合素质。全年民营经济增加值、实缴税金均增长２０％以上。

　　（六）继续加强城市基础设施建设，全面提高城市管理水平

　　以建设“生态园林城市”和“清洁卫生城市”为载体，发挥规划的龙头作用，加快城乡基础设施建设，争创全国优秀卫生城市，加强城市管理和环境保护，提高城市综合竞争力。

　　完善城市功能，进一步改善城市形象。按照产业布局科学、功能分区合理的要求，高水平编制市中心区、阜蒙县城、新邱区“三位一体”发展规划，完善市区建设改造的详细规划设计。发展城市公共交通，以高速公路、通村油路和北环路建设为重点，完善城乡路网建设。沈彰、阜铁、阜朝三条高速公路路面工程完工，开工建设彰武至通辽高速公路。以棚户区改造为契机，加快市区南部地区改造步伐，全面实施孙家湾等集中连片棚户区改造工程。启动采煤沉陷区治理二期工程。大力推进地下管网改造建设，提高供热、供水、供气的安全可靠性，增强城市生产生活保障能力。新增绿地１００万平方米，绿化覆盖率突破４０％。启动九营子河城市段治理工程。

　　发扬人民城市人民建、人民城市人民管的优良传统，深入开展“城市建设管理提高年”活动，共同建设美好家园。完善社会管理，推行城市网格化管理模式，建立科学高效的城市管理机制。严格实施基础设施和公用设施管护制度，提高设施完好率。加强住宅小区环境综合整治，突出抓好物业管理工作，完善物业管理办法，建立社区物业分类管理模式，重点抓好棚改新区物业管理和未封闭小区的环境治理工作。深入开展市容卫生、交通秩序、环境污染等专项整治，切实改善市民生活环境。

　　推进资源节约型、环境友好型城市建设。科学规划和利用土地资源，合理开发矿产资源，实现可持续发展。树立全社会水资源危机意识，加强水资源开发、利用、管理和保护，积极探索和推进水务管理新机制，加快中水回用工程建设，提高水资源利用率，全面建设节水型城市。加快城市生态建设，完成海州矿地质灾害治理一期工程。以治理海州矿烟尘污染为重点，控制工业烟尘排放，取缔小锅炉，减少居民燃煤，改善大气环境，实现生态环境的明显好转。让群众喝上洁净的水，呼吸新鲜的空气。

　　（七）把“民生工程”作为建设和谐社会的重要内容，着力解决关系人民群众利益的突出问题[Page]

　　从解决人民群众最现实、最关心、最直接的问题入手，认真组织实施“民生工程”。大力发展劳动密集型产业、服务业和中小企业，多渠道、多方式增加就业岗位。以稳定就业为目标，落实促进就业的各项政策，加强创业培训和再就业培训，全力做好零就业家庭、棚户区改造回迁居民、“４０５０”人员再就业工作，实现实名制就业５万人。依法加大企业参加社会保险的监管力度，扩大养老、医疗、失业、工伤等各项社会保险的覆盖面，加强社保资金的管理和监督。大力发展社会福利事业和慈善事业，建立城乡医疗救助、住房援助和应急救助等长效机制。完善低保医疗救助和未参保国有困难企业退休人员医疗保险制度。积极开展农村养老保险试点。做好农民工医疗保险工作，开展诚信企业、诚信经理评选活动，维护农民工合法权益。加大住房公积金贷款发放力度，满足城镇中低收入家庭购房需求。建立健全食品、药品和蔬菜质量监测体系，确保人民群众生命健康安全。发展老龄事业。保障残疾人合法权益。加大扶贫力度，完善扶贫机制。健全收入分配机制，促进社会公平正义。让全市人民共享改革发展成果。

　　全年为人民群众办１０件实事。（１）实施５万平方米以下、１万平方米以上集中连片棚户区改造工程。（２）启动建设五龙和高德两座公铁立交桥。（３）对市区９条街巷路和城市道路进行改造。（４）搞好通村油路工程，完成路基改造２６９公里，铺设黑色路面１５６公里。（５）在棚改新区建立６个帮扶站，对困难群众进行“一站式”帮扶。（６）为１０００户居住土坯房的农村灾民（低保户）援建新房。（７）完成１０００个２０户以上自然村通有线电视工作。（８）深化新型农村合作医疗制度，农业人口参合率达到９０％以上。（９）改造维修城乡校舍５万平方米，建设九年寄宿制学校２所。（１０）对６０个重点贫困村实施整村扶贫开发。

　　（八）统筹经济社会发展，推进社会事业全面进步

　　进一步完善科技创新体系，加大科技投入力度。加强科技创新服务平台建设，支持企业开展技术攻关，培育和壮大一批高新技术企业。积极推进企业与辽工大、沈阳工大、省农科院等大专院校及科研院所合作，搞好科技项目对接。加强农业新品种、新技术的引进推广。全年高新技术产业产值增长２０％以上。全面实施素质教育，提高教育质量。扎实推进新一轮“普九”工作，加强教师队伍建设，进一步深化普通高中招生制度改革，促进教育公平。继续深化职业教育办学体制和教学体制改革，整合职业教育资源，完善职业教育体系，充分发挥各级各类职业学校在地方职业技术人才培养中的重要作用。实施人才战略，加强科技人才和企业家队伍建设，加快各类人才培养，积极营造尊重劳动，尊重知识，尊重人才，尊重创造的社会环境。继续实施“一村一名大学生”人才培养工程，做好聘请国外专家项目，不断完善市级农业引智成果推广体系。

　　深入挖掘历史文化资源，增加城市文化底蕴。发展文化事业，打造文化产业，进一步完善公益性文化设施建设，积极做好第二批省级非物质文化遗产保护项目申报和省级文物保护单位晋级工作。制定发展规划及相关政策措施，加快发展动漫产业。认真备战省第七届艺术节。举办农民工文化艺术节。整合资源，统筹规划，精心打造“玛瑙之都”。进一步完善疾病预防控制和医疗救治体系建设，提高公共卫生应急反应能力。以降低医疗服务成本、提高医疗服务质量为目标，整合卫生资源，创新医院管理体制。完成阜蒙县医院、彰武县妇幼保健院和２３所乡镇卫生院改扩建工程。健全医疗卫生服务体系，重点加强农村三级卫生服务网络和以社区卫生服务为基础的新型城市卫生服务体系建设，解决群众看病难、看病贵问题。进一步抓好篮球城建设，积极争创第二批“国家高水平体育后备人才基地”。以迎奥运为契机，广泛开展全民健身活动。加强人口与计划生育工作，提高出生人口素质。启动全市第二轮修志工作。做好第二次全国农业普查工作。[Page]

　　（九）以建设“友爱阜新”、“平安阜新”为目标，推进社会主义精神文明建设和民主法制建设

　　以“建设友爱阜新，争做文明市民”为主题，切实抓好社会主义荣辱观、未成年人思想道德和社会公德、职业道德、家庭美德教育，不断提高市民的文明素质。要动员全社会力量，继续实施蓓蕾生命救助、爱心助学、扶贫帮困等一系列“送温暖、献爱心”活动，在全社会形成“团结友爱、乐善好施、解危济困、诚实守信”的良好风尚。

　　拓宽社情民意表达渠道，建立科学有效的利益协调机制，推行领导干部接待群众制度，落实信访责任制，维护群众利益和社会稳定。全面开展“五五”普法工作，依法及时化解社会矛盾和纠纷。认真落实社会治安综合治理各项措施，普遍建立乡镇、社区警民联防机制，构筑有效的社会治安防范体系。推进城市社会管理视频动态监控系统建设，依法严厉打击各类刑事犯罪，增强人民群众的安全感。开展校园及其周边环境专项整治，维护正常的教育教学秩序。加强突发公共事件应急预案体系建设，建立健全分类管理、分级负责、条块结合、属地为主的应急管理体制，形成统一指挥、反应灵敏、协调有序、运转高效的应急管理机制，提高城市危机管理和抗风险能力。坚持安全第一、预防为主、综合治理，层层落实安全生产责任制，切实抓好安全生产和公共安全工作。要突出抓好煤矿安全，切实提高煤矿安全生产管理水平，强化监督，坚决遏制重大事故发生。

　　实施《各级人民代表大会常务委员会监督法》，自觉接受人民代表大会及其常委会的依法监督和人民政协的民主监督，认真办理人大代表议案、建议和政协提案。发挥统一战线的独特优势，加强与民主党派、工商联、无党派人士和人民团体的联系。充分发挥工会、共青团、妇联和科协等组织的作用。以抓好农村基层廉政建设为重点，搞好基层政权建设，坚持依法、公平、公正的原则，认真做好第九届村民委员会换届选举工作。以建设“设施齐全、服务配套、环境优美、管理有序、文明健康”的新型社区为目标，全面加强社区建设。完善社区公共服务，创新社区管理方式，健全就业、社会保障等社区服务体系，鼓励和吸引社会力量兴办社区服务业。继续做好外事、侨务、对台、民防和国家安全工作，巩固双拥工作成果，进一步密切军政军民关系。

　　认真贯彻《民族区域自治法》，加大对民族地区经济社会发展的支持力度，做大做强民族品牌，巩固发展平等、团结、互助、和谐的社会主义新型民族关系。落实党的宗教政策，发挥宗教在促进社会和谐方面的积极作用，依法管理宗教事务。

　　三、切实加强政府自身建设

　　按照转变职能、权责一致、强化服务、改进管理、提高效能的要求，坚持从体制机制入手，积极创新政府工作，切实提高行政能力和管理水平，着力营造有利于经济社会发展的良好环境，更好地肩负起转型振兴和构建和谐阜新的历史使命。

　　转变政府职能，推进政府管理创新。按照政企分开、政事分开、政府与市场中介组织分开的原则，继续深化行政管理体制改革，更加注重履行社会管理和公共服务职能。以发展社会事业和解决民生问题为重点，优化公共资源配置。创新公共服务体制，改进公共服务方式，加强公共设施建设，为全市人民提供更多更好的公共服务。进一步强化市民投诉工作，坚持市长接听公开电话制度，帮助群众排忧解难。坚持科学民主决策，进一步完善公众参与、专家论证和政府决策相结合的决策机制，使政府决策事项符合人民意愿，经得起历史检验。

　　强化行政责任，提高行政效能。按照权责一致的原则，建立健全行政问责制和责任追究制。加大行政效能监察力度，认真开展行政不作为、乱作为的专项治理，提高政府的公信力和执行力。强化政府部门绩效考核，实行政府内部考核与公众评议、专家评价相结合的评估办法，重点考评政府部门的工作质量和效率。狠抓各项工作落实，搞好重大事项的督查督办，建立政府决策落实的绿色通道。继续办好《行风热线》栏目。[Page]

　　坚持依法行政，规范行政执法行为。认真落实《全面推进依法行政实施纲要》，进一步加强执法队伍建设，严格依照法定权限和程序行使权力、履行职责。规范行政执法行为，切实解决多头执法和执法不严等问题。落实行政执法责任制，完善行政层级监督机制，真正做到有权必有责，用权受监督，侵权要赔偿，违法要追究。进一步做好行政复议应诉工作，切实保护广大公民、法人和其它组织的合法权益。

　　改进工作作风，坚持勤政廉政。坚持一切从实际出发，深入研究和探索服务群众、解决问题、推进工作的有效办法。完善政务公开制度，加快电子政务建设，办好政府网站，及时发布公共信息。继续深化行政审批制度改革，减少和规范行政审批事项。进一步完善行政服务中心功能。充分发挥企业投诉中心作用，不断优化以企业为中心的经济发展软环境。健全教育、制度、监督并重的惩治和预防腐败体系，进一步完善权力运行监督机制，强化执法监察和审计监督。依法严肃查处在工程建设、产权交易、政府采购等领域的商业贿赂案件和损害群众利益行为。严格执行工程质量终身责任追究制。

　　加强学习和培训，提高公务员队伍的整体素质。认真贯彻《公务员法》，加强公务员能力和素质建设。公务员特别是领导干部要把学习放在重要位置，坚持与时俱进、学以致用。紧密结合工作实际，完善公务员学习和培训制度。加强公务员思想、政治、作风建设，增强公务员的大局意识、责任意识、创新意识和服务意识，努力打造一支政治坚定、业务过硬、作风扎实、纪律严明、勤政高效的公务员队伍。

　　各位代表，站在新的历史起点上，形势催人奋进，任务光荣艰巨。有党中央、国务院和省委、省政府的关心和重视，有全市各族人民的信任和支持，我们对阜新的未来充满信心！让我们在中共阜新市委的正确领导下，凝聚全市各族人民的智慧和力量，团结一心，锐意进取，奋发图强，共同开创阜新更加美好的明天！
